# ViromeFlowX: a Comprehensive Nextflow-based Automated Workflow for Mining Viral Genomes from Metagenomic Sequencing Data

**DOI:** 10.1099/mgen.0.001202

**Published:** 2024-02-21

**Authors:** Xiaokai Wang, Zhimin Ding, Ying Yang, Lifeng Liang, Yingshuai Sun, Chaojian Hou, Yuning Zheng, Yan Xia, Lixin Dong

**Affiliations:** ^1^​ Department of Biomedical Engineering, City University of Hong Kong, Hong Kong 999077, PR China; ^2^​ 01Life Institute, Shenzhen, PR China

**Keywords:** Nextflow-based, Viral Genomes Mining, ViromeFlowX

## Abstract

Understanding the link between the human gut virome and diseases has garnered significant interest in the research community. Extracting virus-related information from metagenomic sequencing data is crucial for unravelling virus composition, host interactions, and disease associations. However, current metagenomic analysis workflows for viral genomes vary in effectiveness, posing challenges for researchers seeking the most up-to-date tools. To address this, we present ViromeFlowX, a user-friendly Nextflow workflow that automates viral genome assembly, identification, classification, and annotation. This streamlined workflow integrates cutting-edge tools for processing raw sequencing data for taxonomic annotation and functional analysis. Application to a dataset of 200 metagenomic samples yielded high-quality viral genomes. ViromeFlowX enables efficient mining of viral genomic data, offering a valuable resource to investigate the gut virome’s role in virus-host interactions and virus-related diseases.

## Data Summary

This study demonstrates the use of openly available BioProject accession PRJNA557323 raw metagenomic sequence reads as a representative dataset. The ViromeFlowX tool, which can be accessed by all researchers on GitHub at (https://github.com/01life/ViromeFlowX), provides detailed instructions. Besides, two additional publicly available datasets, one consisting of virus-free genomes (https://zenodo.org/records/4297575) [1] and the other containing virus-infected samples (PRJNA698986) [2] are further tested by ViromeFlowX, and all corresponding details and results are available at https://github.com/01life/ViromeFlowX_V1_DATA/tree/main/verified_result.

**Impact Statement**

Gut viruses are a topic of significant interest for their roles in shaping the composition of the gut microbiome and regulating human homeostasis. Mining viral genomes from shotgun metagenomic sequencing data is vital for understanding viral communities, virus-bacterium-host interactions, and disease associations. The current tools for viral genome analysis still exhibit certain constraints. Therefore, we have developed ViromeFlowX, a Nextflow-based, highly flexible, modular, customizable, and easy-to-use automatic monitoring pipeline with various applications and databases. ViromeFlowX seamlessly processes raw reads, assembles viral genomes, and provides taxonomic annotations and gene functional analyses. Validated using real data, the pipeline efficiently reduces analysis time, obtains high-quality viral contigs, and offers comprehensive taxonomic and functional annotations. The pipeline’s user-friendly nature, scalability, and customizable execution make it a potent tool for unravelling the secrets of viral communities, paving the way for breakthroughs in virome research.

## Introduction

The gut microbiome dramatically impacts human health and disease, and its infecting viruses are likely equally important [3–6]. Emerging views suggest that the gut virome plays a vital role in regulating homeostasis and disease progression through interactions with the bacteriome and the human immune system [7]. These studies have revealed significant associations between changes in phage composition and several diseases [8–11], such as metabolic syndrome [12, 13], necrotizing enterocolitis [14], inflammatory bowel disease [15–18], and type two diabetes mellitus [19, 20]. Recent cross-cohort meta-analyses have identified specific gut viral biomarkers, such as phages of Porphyromonas, Fusobacterium, and Hungatella, enriched in colorectal cancer patients, suggesting their potential as treatment targets [21]. Johansen *et al*.’s research has highlighted the gut virome’s impact on longevity. They found that centenarians have a more diverse and slower-working gut virome, which could potentially affect their metabolism [22]. These studies highlight the significance of exploring viral communities founded in metagenomes to understand their effects on human health.

Despite the rapid development of metagenomics techniques and the burgeoning availability of microbial sequencing data, previous research has predominantly focused on bacteria, ignoring the intricate interactions between bacterial and viral communities [23]. However, the advent of shotgun sequencing data offers a valuable avenue for comprehending the dynamic relationship between phages and bacterial ecosystems, eliminating the necessity for viral particle-specific enrichment. While several pipelines have emerged to uncover viral communities from metagenomic data, they still exhibit certain constraints. For instance, ViWrap lacks provisions for quality control and assembly of raw reads, gene prediction, and functional annotation of viral contigs [24]. Similarly, while VirMAP facilitates iterative assembly enhancement and the detection of viral sequences from metagenomic datasets, it does not encompass gene prediction and functional annotation of viral contigs [25].

In response to these challenges and to optimize the analysis of viral genomes within metagenomic data, we have developed ViromeFlowX, a Nextflow-based pipeline. This pipeline efficiently handles viral genomes, from processing raw next-generation sequencing reads to providing viral taxonomic annotation and gene functional analysis. Leveraging the power of Nextflow [26], ViromeFlowX guarantees separate module execution, efficient parallel processing, error resilience, and traceable execution history. It effortlessly adapts to various environments, including local machines, high-performance computing, and cloud infrastructures, enabling swift pipeline development and parameter customization.

ViromeFlowX leverages the capabilities of Virfinder [27], Virsorter2 [2], and robust viral identification tools, to efficiently detect viral contigs. These contigs undergo taxonomic classification through four distinct strategies. The pipeline calculates the abundance of viral contigs, gene functional levels, and a seven-level hierarchical taxonomy. Kraken2 [28], a swift taxonomic classifier, is also integrated for viral taxonomy identification and quantification to avoid overlooking low-abundance viruses. The user-friendly interface, automated task tracking, and well-organized result directory minimize usability barriers. Using ViromeFlowX, researchers can harness its capabilities to effectively mine virus data, deepen our understanding of viral populations, and elucidate the intricate relationship between human health and viruses.

## Methods and Results

### ViromeFlowX workflow

**Fig. 1.**
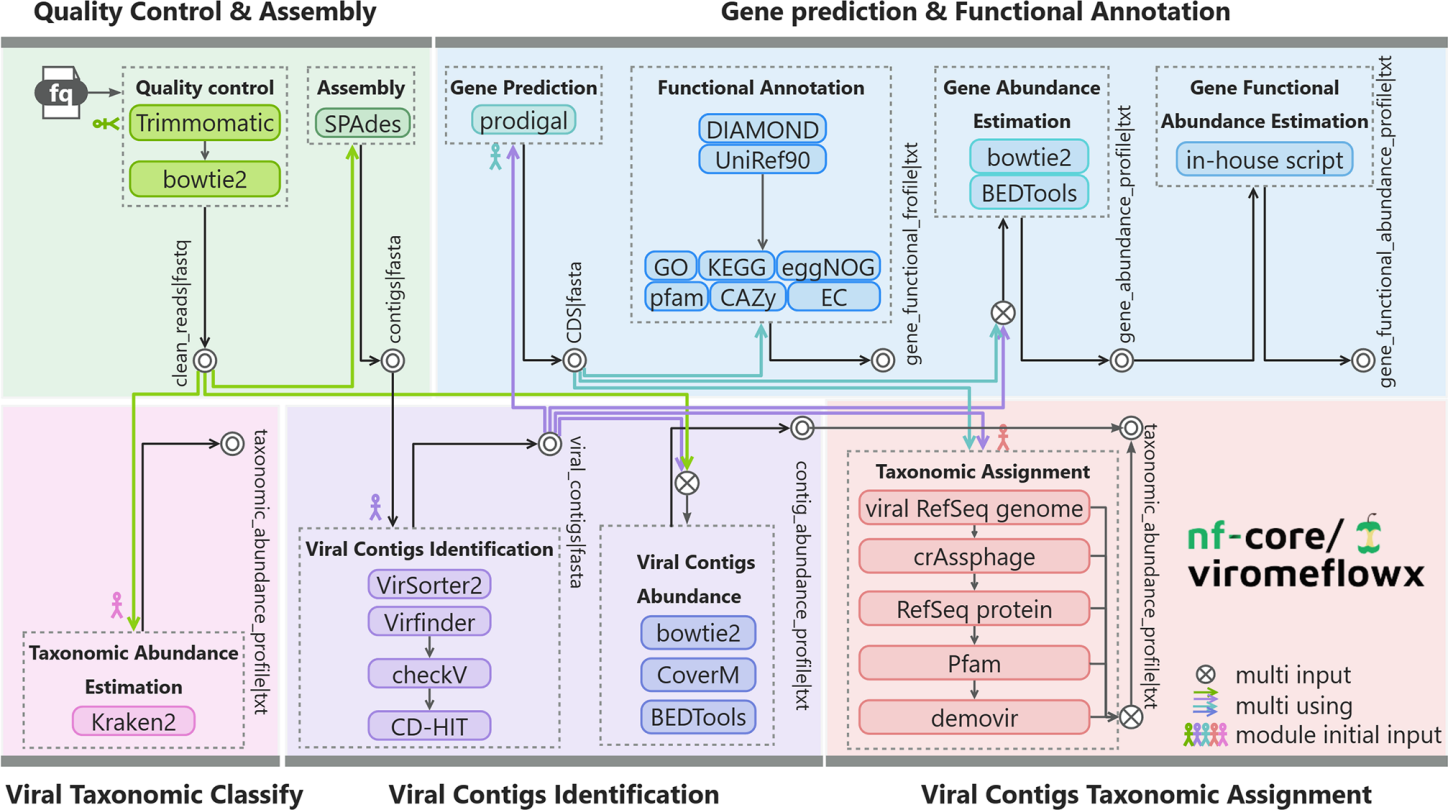
Schematic overview of the ViromeFlowX. The general flowchart of ViromeFlowX is colour-coded by modules. Coloured lines signify that the file flow will serve as input for multiple subprograms. Intersecting circles indicate that the subprogram requires multiple input files, while concentric circles indicate that the programme at this point has published result files.

ViromeFlowX is an easy-to-instal, user-friendly, parameter-clear, and highly scalable virome analysis tool. It consists of various software and is divided into five modules, each focusing on an important step in the analysis process. With a single command, it orchestrates quality control and genome assembly (1), identification and taxonomic assignment of viral contigs (2) and (3), gene prediction and functional annotation (4), and viral taxonomic classification via Kraken2 (5) (Fig. 1). Generally, the software employs default parameters, while pivotal parameters can be conveniently accessed and modified within the Nextflow config file. This modular arrangement simplifies parameter manipulation, enhancing user control. Resource allocation for each software is optimized, and outcomes are thoughtfully organized within the default 'work' output directory. This thoughtful structuring eases user examination, circumventing the necessity of delving into intricate intermediate files. As a result, the threshold for using the workflow is greatly reduced.

ViromeFlowX offers two execution modes. The recommended approach launches the complete pipeline via a single command, such as ‘nextflow run nf-core-virome --input sample.csv --outdir <OUTDIR>’ . However, users seeking tailored analyses can selectively omit certain modules by appending ‘--skip_<module name>’ to the command. Notably, ViromeFlowX encapsulates all essential tools and third-party dependencies within a conda environment, harmoniously coexisting with existing programmes. This environment harnesses Python version 3.10.8 and aligns with the R statistical package version 4.2.2. A comprehensive inventory of software and databases leveraged by ViromeFlowX can be found in Table 1, backed by meticulous benchmarking informed by published literature.

**Table 1.** Comprehensive list of third-party tools and databases used in ViromeFlowX

Software or database	Version	Summary	Links
**Software**			
Trimmomatic	0.36	Read trimmer for Illumina NGS data	https://github.com/timflutre/trimmomatic
Bowtie2	2.5.0	A fast and sensitive gapped read aligner	https://github.com/BenLangmead/bowtie2
SPAdes	3.11	*De novo* assembly of genome sequencing data	https://github.com/ablab/spades
VirSorter2	2.1	Pipeline to identify viral sequences from (meta)genomic data	https://github.com/jiarong/VirSorter2
VirFinder	1.1	A novel k-mer-based tool for identifying viral sequences from assembled metagenomic data	https://github.com/jessieren/VirFinder
CD-HIT	4.8.1	Accelerated clustering of the next-generation sequencing data	http://cd-hit.org.Contact
checkV	1.0.1	Assesses the quality and completeness of metagenome-assembled viral genomes	https://bitbucket.org/berkeleylab/CheckV
blastn, blastp	2.11.0	A web interface for sequence similarity search	http://www.ncbi.nlm.nih.gov/blast
taxonkit	0.7.2	A practical and efficient NCBI taxonomy toolkit, also supports creating NCBI-style taxdump files for custom taxonomies like GTDB/ICTV	https://github.com/shenwei356/taxonkit
CoverM	0.6.1	Read coverage calculator for metagenomics	https://github.com/wwood/CoverM
bedtools	2.30.0	A flexible suite of utilities for comparing genomic features	https://github.com/arq5x/bedtools2
Prodigal	2.6.3	Prokaryotic gene recognition and translation initiation site identification	https://github.com/hyattpd/Prodigal
Prokka	1.14.6	Rapid prokaryotic genome annotation	https://github.com/tseemann/prokka
diamond	2.0.6	Sensitive protein alignments at tree-of-life scale	https://github.com/bbuchfink/diamond
demovir		Taxonomic classification of viruses at Order and Family level	https://github.com/feargalr/Demovir
**Database**			
uniref90	01/2019	A database consisting of clustered sets of sequences from UniProtKB and selected UniParc records	http://www.uniprot.org/uniref
NCBI RefSeq sequence	10/6/2021	A comprehensive, integrated, non-redundant, well-annotated set of reference sequences including genomic, transcript, and protein	https://www.ncbi.nlm.nih.gov/refseq/
crAssphage	NC_024711.1	The most abundant virus in the human gut	https://www.sciencedirect.com/science/article/pii/S1931312818305249
eggNOg	5.0	A hierarchical, functionally and phylogenetically annotated orthology resource	http://eggnog5.embl.de/#/app/home
pfam	11/1/2021	A database of conserved protein families and domains.	http://pfam.xfam.org/

### Reads quality control and genome assembly

In the initial stage of our analysis, we applied Trimmomatic [29], a tool utilized for filtering low-quality reads, reads containing adapters, or reads that are too short. Subsequently, our custom-built in-house kit, a tool used to quality control developed by ourselves, selectively removed host genome sequences identified by Bowtie2 [30]. This remarkable kit proved to be more efficient and saved valuable resources, surpassing the capabilities of the KneadData software (https://github.com/biobakery/kneaddata). Each metagenomic sample underwent individual contig assembly using SPAdes [31] with the ‘-meta’ option and default parameters. Contigs below 1 kb were excluded from further analysis, ensuring a focus on more substantial genetic components.

### Viral contig extracted from raw assemblies

The extraction of viral contigs from metagenomic data was a meticulous process achieved through the use of two cutting-edge tools: VirSorter2 [31] and Virfinder [27]. VirSorter2 seamlessly integrates sequence similarity and distinctive viral-like features to classify entire contigs within the dataset. Simultaneously, Virfinder relies on the nuanced analysis of k-mer signatures to pinpoint viral contigs exclusively. The union of the results obtained from both tools were then merged to ensure comprehensive coverage of potential virus sequences. To ensure the quality and reliability of the identified viral genomes, we employed CheckV [32], a powerful tool that assesses various aspects of single-contig viral genomes. This process entails detecting host contaminations in integrated proviruses, gauging the completeness of genome fragments, and recognizing fully closed genomes. The amalgamation of CheckV results provided a comprehensive view of the potential viruses harboured within each sample. We employed CD-HIT [33], a highly efficient clustering algorithm that curates a non-redundant collection of virus contig sequences to streamline the analysis. This step significantly optimized the analytical process, especially when dealing with multiple samples, by reducing redundancy and conserving computational resources. Notably, this approach alleviated resource demands for subsequent analysis, potentially lowering the threshold for large-scale studies. With the aim of quantification, we determined the contigs' coverage using the complementary capabilities of CoverM (https://github.com/wwood/CoverM) and BEDTools [34]. Further refinement involved a custom Perl script, engineered to convert coverage values into an abundance profile, quantified as Reads Per Kilobase per Million mapped reads (RPKM). This meticulous process underpinned the accuracy and robustness of our subsequent viral analysis.

### Gene prediction and functional annotation

To uncover the genetic structure of viral contigs, we initiated gene prediction by utilizing Prodigal [35], which expertly identifies open reading frames (ORFs). These ORFs were subsequently aligned with precision to the UniProt Reference Clusters (UniRef) 90 database (release 01/2019) [36] using DIAMOND [37], a robust alignment tool. For exact results, our Perl script identified the best match based on the results from DIAMOND. The selection criteria were strict. Only records with a coverage of over 80 % and an identity of more than 50 % were included as annotation records. We continued transforming UniRef90 database items into actionable annotation insights for Gene Ontology (GO) [38, 39], EGGNOG [40], Kyoto Encyclopaedia of Genes and Genomes (KEGG) [41], PfamA [42], EC [41], and the Carbohydrate-Active EnZymes database (CAZy) [43] databases. In-house scripts were used to map UniRef90 entries to these annotations. The Bowtie2 [30] tool and BEDTools [34] have proven helpful in measuring the number of genes present. Bowtie2 efficiently quantified gene content, while bedtools accurately gauged the coverage of each cluster across multiple samples. The resulting gene abundance table underwent normalization via the RPKM method, yielding a comprehensive gene abundance profile table. Our in-house R script was employed to enhance further functionality to convert UniRef90 gene abundance into actionable profiles within the functional database. This intricate gene prediction and available annotation process greatly enriched the contextual understanding of the viral contigs and their potential roles.

### Viral taxonomic classification

**Fig. 2.**
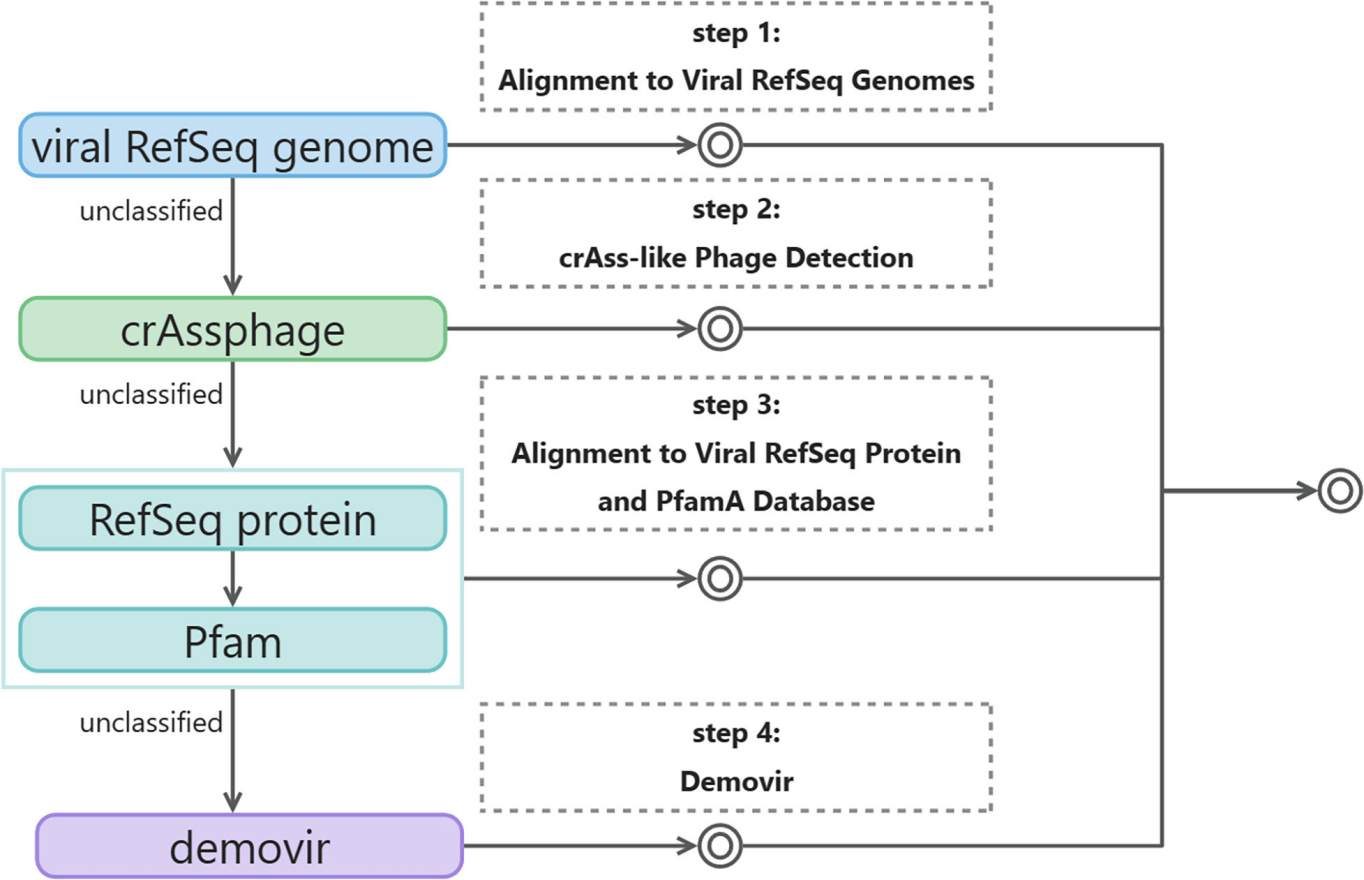
Workflow of four-step viral taxonomic classification of viruses. Extracted viral contigs were sequentially assigned to a seven-level hierarchical taxonomy. The flowchart is colour-coded by steps, concentric circles indicate that the programme at this point has published result files.

Building upon K. Fujimoto *et al*.’s [44] viral taxonomic classification method, we refined and enhanced viral contig annotations to better understand their biological significance. We have created viral genome and protein databases to classify the viral contigs. The process of virus classification annotation consists of four sequential steps (Fig. 2). First, we aligned the contigs with viral RefSeq genomes [45]. If the contigs did not align, we conducted further analysis using crAss-like phage detection [46] to try and obtain classification information. For contigs that were still not classified, we attempted to identify similar viral proteins at the protein level to infer taxonomic classification. Lastly, contigs that showed no similarity to known proteins were annotated using Demovir as a final step. Here are the specific methods used for each of the four steps:

#### Step 1: Alignment to Viral RefSeq Genomes

We harnessed the viral RefSeq sequences (downloaded on 10 June 2021) [45], encompassing 14 679 genomes and 546 060 proteins from NCBI. Leveraging blastn [47], we aligned viral contig sequences against these genomes, employing an E-value threshold of 1e-10. The taxonkit [48] coupled with the NCBI taxonomy pinpointed the lowest common ancestor (LCA) based on the top five hits, thus attributing classification to the contigs.

#### Step 2: crAss-like Phage Detection

The annotation of contig ORFs facilitated crAss-like phage detection. We employed blastp [37] to query amino acid sequences of prototypical crAssphage genetic signatures (p-crAssphage, NC_024711.1) against contig ORFs, using an E-value cutoff of 1e-10.

#### Step 3: Alignment to Viral RefSeq Protein and PfamA Database

Predicted ORFs lacking classification in previous steps underwent a blastp search against the viral RefSeq protein database from Step 1, using an E-value threshold of 1e-10 and a bitscore exceeding 50. Leveraging phage structural proteins in the PfamA [42] annotation, viral contigs were classified based on specific proteins. This PfamA family included phage tail protein, phage tail sheath protein, or Microviridae capsid protein gene, attributing contigs to Caudovirales, Myoviridae, or Microviridae.

#### Step 4: Demovir

Contigs not classified earlier received family-level annotations via the Demovir script (https://github.com/feargalr/Demovir) with default parameters and database. This script hunted for homologies in amino acid sequences between contig-encoded proteins and a viral subset of the TrEMBL database. Taxonomic assignment employed a voting approach.

These four steps were ultimately amalgamated into comprehensive taxonomic classifications for each contig. The results, encompassing taxonomic levels from phylum to species, were exported in BIOM-format [49]. Kraken2 [28] was harnessed using a viral database to address low-abundance viruses, which are often overlooked. Bringing Kraken2 into our workflow enabled an alternate perspective on viral sample composition, thus broadening researchers' insights into viral diversity.

### Results organization

The final output directory is meticulously organized, ensuring easy access to comprehensive results. Here’s an overview of the layout:

#### 01. QC:

This section showcases the outcomes of the read quality control step. Inside, you'll discover the clean reads for each sample, formatted in fastq. These reads are the result of Trimmomatic application.

#### 02. assembly:

Here, the results of viral assemblies are stored in the directory. This folder contains the contig fasta files for each sample.

#### 03. identify:

This dedicated folder encompasses distinct sub-folders for VirFinder and VirSorter2 outcomes. Moreover, a consolidated folder brings together results from both runs.

#### 04. predict:

This directory contains a refined subset of viral contigs post-CD-HIT deduplication. It also houses intermediary CD-HIT results. Expect to find Prodigal gene predictions on deduplicated contigs enriched with nucleotide sequences, protein sequences, and GFF format annotations.

#### 05. classify:

This directory is the result of viral taxonomic classification insights. It incorporates the results from the four classification steps, leading to the final merging of classification outcomes.

#### 06. abundance:

Abundance profiling at both contig and gene levels. Every sample has unique characteristics, and when all the samples are combined, it enhances the dataset even more.

#### 07. functional:

Annotations abound in this repository, featuring a symphony of results against uniref90 and GO, EggNOG, KEGG, PfamA, EC, and CAZy databases.

#### 08. profile:

Witness the profiles come to life! This folder contains abundance profiles at various taxonomic levels, spanning viral entities and functional databases. The Kraken results are also in this folder.

#### pipeline_info:

This directory encapsulates Nextflow’s insights into the workflow run. It offers a window into job execution status and the detailed utilization of computing resources, guiding users on the journey.

This meticulous arrangement empowers you to seamlessly navigate through the intricacies of your results, enhancing your analytical prowess.

### Performance excellence

Leveraging nf-core’s adept management of task execution and optimal allocation of computing resources, ViromeFlowX works well across datasets of varying dimensions. We delved into three metagenomic samples through meticulous evaluation, each harbouring an average raw data size of 6G. Employing a robust elastic computing cluster boasting 32 CPUs and a generous 64G memory capacity, our analysis executed seamlessly, culminating in completion within a mere 6 h.

As we took on the task of testing bulk metagenomes, we discovered the increased strength and reliability of ViromeFlowX. With a comprehensive study of 200 human gut microbiome samples [50], each with an average of 6G sequencing data, totaling 658G, we conducted this analysis using the Amazon Cloud platform, fortified with a CPU limit 3200. The journey concluded within 3 days and 12 h, underlining the workflow’s efficiency and batch-processing prowess. This impressive performance slashes analysis time and grants analysts respite from continuous job monitoring.

Beyond streamlined analysis, ViromeFlowX offers a panoramic view of metagenomic virome analysis. From the inception of read quality control to the culmination of taxonomy abundance assignment and multi-faceted database annotation, the workflow’s coverage is comprehensive. Table 2 illustrates that ViromeFlowX may presently lack modules for viral host prediction. Its advantage lies in taxonomy annotation and abundance profiling, surpassing other commonly employed metagenomic virome analysis suites. With an elegantly structured workflow and an expansive spectrum of functionalities, ViromeFlowX is a beacon of cutting-edge technology for the meticulous dissection of viral communities embedded within metagenomic landscapes.

**Table 2.** Comparison of ViromeFlowX functionality with commonly used metagenome assembly and binning pipelines

Functionality	nf-core/ViromeFlowX	nf-core/viralrecon	VirWrap
Support Nanopore Sequencing data	×	√	×
QC	√	√	×
Assembly	√	√	×
Taxonomic Abundance Estimation	√	×	×
Virus identification and annotation	√	×	√
Virus quality characterization	√	×	√
Functional Annotation	√	×	×
Virus binning and clustering	×	×	√
Virus taxonomy classification	√	×	√
Virus host prediction	×	×	√
Variant calling	×	√	×

### Validation and visualization on real dataset

**Fig. 3.**
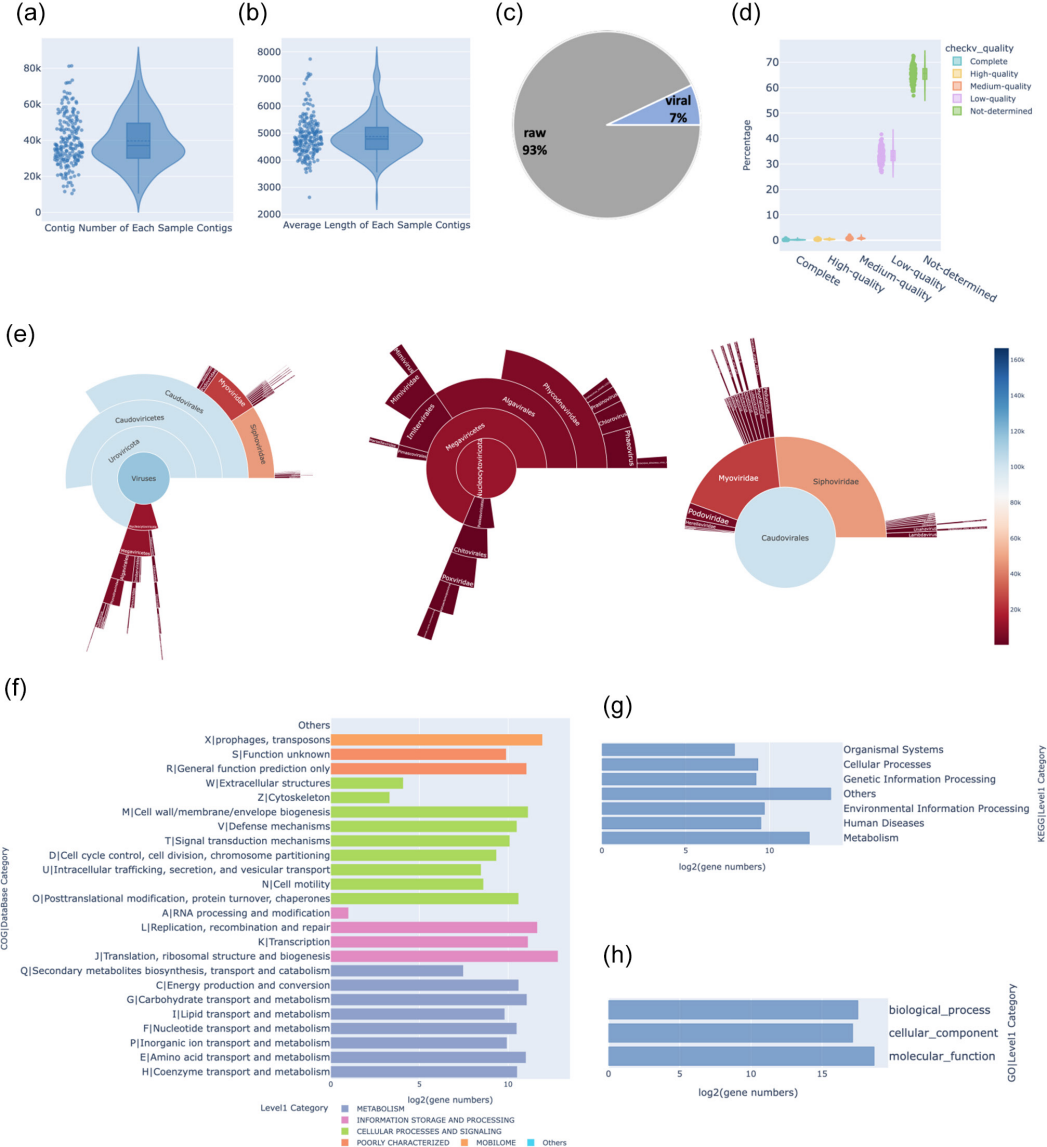
Visualisations of virus statistics. The violin chart (**a**) showcases contig distribution across samples, while (**b**) portrays average contig length. The pie chart (**c**) visually encapsulates viral contig presence. The violin plot (**d**) reveals the quality distribution of viral contigs. The sunburst chart (**e**) maps viral taxonomy and highlights Nucleocytoviricota and Uroviricota. Bar charts (**f–h**) depict gene counts in COG, KEGG, and GO databases.

To thoroughly validate the efficacy of ViromeFlowX, we conducted an in-depth analysis of a dataset comprised of 200 published faecal metagenomes (Table S1, available in the online version of this article) from healthy individuals in Hong Kong. These data were assembled into 7 930 827 raw contigs, with an average assembly of 39 654 contigs per sample, and a mean contig length of 4871 kb (Fig. 3a, b; Tables S2 and S3). With the integration of VirSorter and Virfinder, our analysis identified 598 566 viral contigs, yielding a recall rate of 7.54 % (Fig. 3c and Table S4). These viral contigs were refined to 3007 per sample, boasting an average length of 3477. A rigorous CheckV evaluation highlighted that 65.71 % of the contigs could not be conclusively determined. Among the remainder, 33.06 % were categorized as low-quality, while the prevalence of medium-quality, high-quality, and complete contigs stayed below 1 %. Importantly, our analysis solidified 1066 complete and 2340 high-quality viral contigs (Fig. 3d and Table S5). During virus classification annotation, the contigs were assigned to 14 phyla, 21 classes, and 53 families (Fig. 3e and Table S6). A notable domain class emerged as Caudoviricetes, with the leading genera or species affiliated with Nucleocytoviricota (Fig. 3f) and Uroviricota (Fig. 3G) phyla. Then, Prodigal was utilized to predict open reading frames (ORFs). A total of 1 548 275 ORFs were categorized into 267 525 gene clusters, incorporating alignment thresholds of 80 % for length and 50 % for identity. Our analysis delved into virome functions, anchored in COG, KEGG and GO databases (Tables S7–S9), complete with corresponding gene count fractions (Fig. 3f–h). These visually impactful findings provided an instinctive grasp of the quantified characteristics of the viral community, virus quantities, and gene counts of various databases.

## Discussion

ViromeFlowX is a Nextflow pipeline that streamlines the process of mining viral genomes from metagenomic data. What sets ViromeFlowX apart from other pipelines is its ability to not only assemble viral genomes but also perform comprehensive viral taxonomy classification and abundance estimation using state-of-the-art taxonomy classifiers. This feature-rich pipeline is specifically designed to handle large metagenomic samples and consistently deliver reproducible results, ensuring reliable outcomes for diverse research questions and experimental setups.

By leveraging Nextflow, ViromeFlowX automates the execution of analyses with built-in error recovery and checkpoint resumption capabilities. The visual run report provides users with a transparent view of the workflow execution process, promoting ease of interpretation and monitoring.

The modular organization of ViromeFlowX allows users to customize their analyses by skipping unnecessary steps and focusing on specific functionalities. This flexibility empowers researchers to tailor the pipeline to their unique requirements and compare different settings effectively.

While ViromeFlowX represents a significant advancement in viral genomic analysis, continuous maintenance and development are essential to keep pace with evolving analytical approaches. As part of future improvements, the upcoming version, ViromeFlowX2, is poised to introduce additional features such as binning techniques [51] and in-depth analysis of viral genomes, including host prediction, further enhancing its utility and applicability. ViromeFlowX2 is committed to achieving a custom expandable virus database, allowing users to add new knowledge of virus data to prepare for the potential emergence of new mutated viruses in the future.

One aspect that requires attention in the current code is the use of intermediate ‘work’ directories, leading to a fourfold increase in storage requirements for running a single sample. This storage inefficiency arises from Nextflow’s retention of all result files, including temporary ones, unless manually deleted. To mitigate this issue, the upcoming ViromeFlowX version will implement an automated mechanism to remove unnecessary intermediate files, optimizing storage usage and streamlining the overall workflow execution process.

## Conclusion

ViromeFlowX is a robust and user-friendly solution that empowers researchers to investigate viral communities in metagenomic datasets. Its automation, adaptability, and integration of advanced tools make it a valuable resource for viral genomic data analysis. By using ViromeFlowX, the scientific community can better understand the complex relationships between viruses and their hosts, leading to discoveries and advancements in virome research.

## Supplementary Data

Supplementary material 1
